# Poly[diaqua­bis­(nitrato-κ^2^ *O*,*O*′)bis­(1,10-phenanthroline-κ^2^ *N*,*N*′)-μ_3_-succinato-dicadmium]

**DOI:** 10.1107/S1600536812025287

**Published:** 2012-06-13

**Authors:** Hong-Jin Li, Zhu-Qing Gao, Jin-Zhong Gu

**Affiliations:** a School of Chemical and Biological Engineering, Taiyuan University of Science and Technology, Taiyuan 030021, People’s Republic of China; b College of Chemistry and Chemical Engineering, Lanzhou University, Lanzhou 730000, People’s Republic of China

## Abstract

In the title coordination polymer, [Cd_2_(C_4_H_4_O_4_)(NO_3_)_2_(C_12_H_8_N_2_)_2_(H_2_O)_2_]_*n*_, the Cd^II^ ion is seven-coordinated within a distorted penta­gonal–bipyramidal O_5_N_2_ environment. The succinate anions, located on an inversion centre, adopt a bis-monodentate bridging mode, leading to the formation of rods along [100]. The rods are connected by O—H⋯O hydrogen bonds between the coordinating water mol­ecules and nitrate O atoms of adjacent rods; the same type of hydrogen bonds are also observed between water and carboxyl­ate O atoms within the rods. π–π stacking inter­actions with a minimum plane-to-plane separation of 3.462 (2) Å occur between phenanthroline ligands.

## Related literature

For the structures and properties of other cadmium coordination compounds, see: Montney *et al.* (2007 ▶); Li *et al.* (2011 ▶).
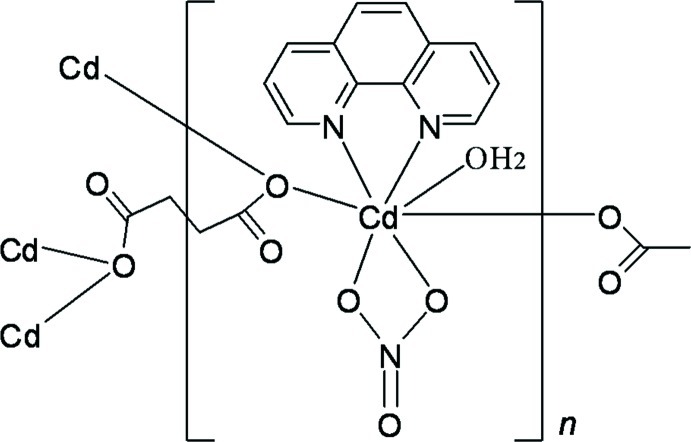


## Experimental

### 

#### Crystal data

[Cd_2_(C_4_H_4_O_4_)(NO_3_)_2_(C_12_H_8_N_2_)_2_(H_2_O)_2_]
*M* *_r_* = 861.33
Triclinic, 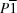

*a* = 7.7349 (13) Å
*b* = 9.4467 (16) Å
*c* = 11.2063 (18) Å
α = 102.284 (2)°
β = 106.059 (2)°
γ = 107.003 (2)°
*V* = 713.0 (2) Å^3^
*Z* = 1
Mo *K*α radiation
μ = 1.57 mm^−1^
*T* = 110 K
0.40 × 0.34 × 0.32 mm

#### Data collection

Bruker APEXII CCD diffractometer
Absorption correction: multi-scan (*SADABS*; Bruker, 2008 ▶) *T*_min_ = 0.572, *T*_max_ = 0.633
5357 measured reflections
2617 independent reflections
2333 reflections with *I* > 2σ(*I*)
*R*_int_ = 0.021

#### Refinement

*R*[*F* ^2^ > 2σ(*F* ^2^)] = 0.026
*wR*(*F* ^2^) = 0.086
*S* = 1.10
2617 reflections
223 parameters
2 restraints
H atoms treated by a mixture of independent and constrained refinement
Δρ_max_ = 0.82 e Å^−3^
Δρ_min_ = −0.74 e Å^−3^

### 

Data collection: *APEX2* (Bruker, 2008 ▶); cell refinement: *SAINT* (Bruker, 2008 ▶); data reduction: *SAINT*; program(s) used to solve structure: *SHELXS97* (Sheldrick, 2008 ▶); program(s) used to refine structure: *SHELXL97* (Sheldrick, 2008 ▶); molecular graphics: *XP* in *SHELXTL* (Sheldrick, 2008 ▶) and *DIAMOND* (Brandenburg, 2006 ▶); software used to prepare material for publication: *publCIF* (Westrip, 2010 ▶).

## Supplementary Material

Crystal structure: contains datablock(s) I, global. DOI: 10.1107/S1600536812025287/wm2640sup1.cif

Structure factors: contains datablock(s) I. DOI: 10.1107/S1600536812025287/wm2640Isup2.hkl

Additional supplementary materials:  crystallographic information; 3D view; checkCIF report

## Figures and Tables

**Table 1:** Hydrogen-bond geometry (Å, °)

*D*—H⋯*A*	*D*—H	H⋯*A*	*D*⋯*A*	*D*—H⋯*A*
O1*W*—H1*B*⋯O3^i^	0.86 (1)	1.93 (1)	2.786 (5)	174 (5)
O1*W*—H1*A*⋯O2^ii^	0.86 (1)	1.88 (2)	2.716 (4)	166 (5)

Symmetry codes: (i) 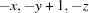; (ii) 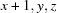.

**Fig. 1.**
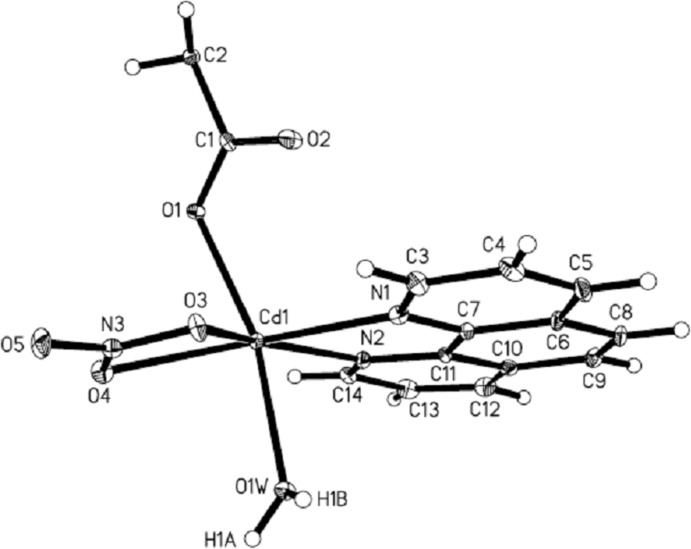
The asymmetric unit of the structure of (I) with displacement ellipsoids drawn at the 30% probability level.

**Fig. 2.**
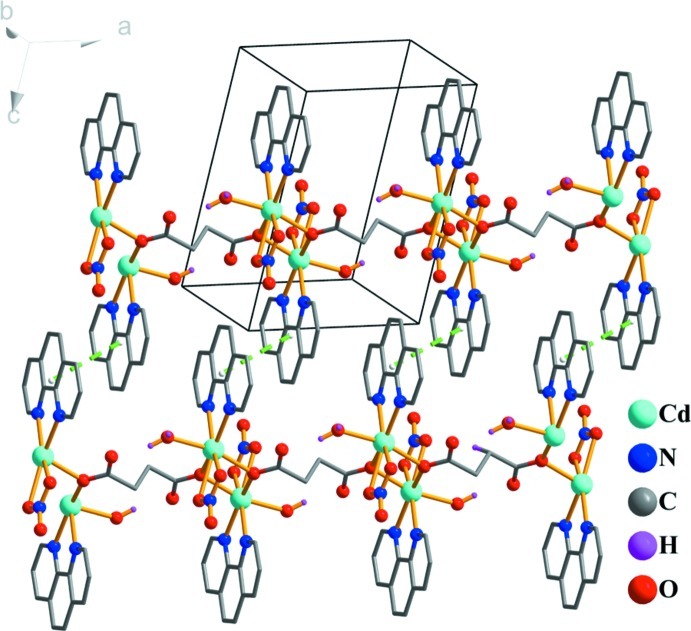
View approximately along the b axis, showing the rods parallel to [100] in the title complex. The green dashed lines represent π—π stacking interactions.

**Fig. 3.**
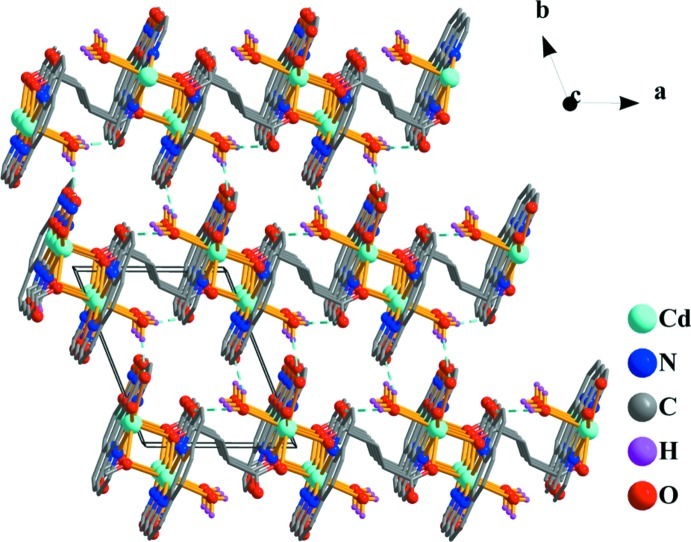
View along the c axis, showing the three-dimensional supramolecular network. Blue dashed lines represent O—H···O hydrogen bonds.

## supplementary crystallographic information

### Comment

Cadmium(II) coordination compounds have been increasingly studied owing to their interesting physical and chemical properties (Montney *et al.*, 2007; Li *et al.*, 2011). In order to extend our knowledge in this field, we investigated the structure of the cadmium(II) title compound, [Cd_2_(C_4_H_4_O_4_)(H_2_O)_2_(C_12_H_8_N_2_)_2_(NO_3_)_2_], (I).

The asymmetric unit of (I) (Fig. 1) contains one Cd^II^ ion, half of a succinate anion, one nitrate anion, one coordinating water and one 1,10-phenanthroline ligand. The Cd^II^ cation is seven-coordinated by five O atoms from two succinate anions, one water molecule and a chelating nitrate anion, and two N atoms from a 1,10-phenanthroline molecule. The coordination geometry around Cd^II^ might be described as a distorted pentagonal bipyramid. The succinate anions adopt a bis-monodentate bridging coordination mode, generating rods along [100]. Adjacent rods are connected by O—H···O hydrogen bonds and aromatic π—π stacking interactions [minimum plane-to-plane separation of 3.462 (2) Å] to generate a three-dimensional network (Figs. 2,3).

The Cd–N and Cd–O bond lengths are 2.358 (4)–2.360 (4) Å and 2.253 (3)–2.511 (3) Å, respectively, which are comparable to those reported for other Cd(II)–O and Cd(II)–N donor complexes (Montney *et al.*, 2007; Li *et al.*, 2011).

### Experimental

A mixture of Cd(NO_3_)_2_^.^4H_2_O (0.155 g, 0.50 mmol), succinic acid (0.029 g,0.25 mmol), 1,10-phenanthroline (0.10 g, 0.5 mmol), NaOH (0.02 g,0.5 mmol), and water (10 ml) was stirred at room temperature for 15 min, and then sealed in a 25 ml Teflon-lined, stainless-steel Parr bomb. The bomb was heated at 433 K for 3 days. Upon cooling, the solution yielded single crystals of the title complex in *ca* 65% yield. Anal.Calcd for C_14_H_12_N_3_O_6_Cd: C,39.04; H, 2.81; N, 9.76. Found: C, 38.73; H, 3.14; N, 9.63.

### Refinement

The coordinating water H atoms were located in a different Fourier map and refined with distance constraints of O—H = 0.83 (3) Å. The carbon-bound H atoms were placed in geometrically idealized positions, with C–H= 0.93 Å, and constrained to ride on their respective parentatoms, with *U*iso(H) = 1.2 *U*eq(C).

### Crystal data

**Table:**

[Cd_2_(C_4_H_4_O_4_)(NO_3_)_2_(C_12_H_8_N_2_)_2_(H_2_O)_2_]	*Z* = 1
*M**_r_* = 861.33	*F*(000) = 426
Triclinic, *P*1	*D*_x_ = 2.006 Mg m^−^^3^
Hall symbol: -P 1	Mo *K*α radiation, λ = 0.71073 Å
*a* = 7.7349 (13) Å	Cell parameters from 4041 reflections
*b* = 9.4467 (16) Å	θ = 2.4–25.5°
*c* = 11.2063 (18) Å	µ = 1.57 mm^−^^1^
α = 102.284 (2)°	*T* = 110 K
β = 106.059 (2)°	Block, colorless
γ = 107.003 (2)°	0.40 × 0.34 × 0.32 mm
*V* = 713.0 (2) Å^3^	

### Data collection

**Table:**

Bruker APEXII CCD diffractometer	2617 independent reflections
Radiation source: fine-focus sealed tube	2333 reflections with *I* > 2σ(*I*)
Graphite monochromator	*R*_int_ = 0.021
φ and ω scans	θ_max_ = 25.5°, θ_min_ = 2.0°
Absorption correction: multi-scan (*SADABS*; Bruker, 2008)	*h* = −9→9
*T*_min_ = 0.572, *T*_max_ = 0.633	*k* = −11→11
5357 measured reflections	*l* = −13→13

### Refinement

**Table:**

Refinement on *F*^2^	Primary atom site location: structure-invariant direct methods
Least-squares matrix: full	Secondary atom site location: difference Fourier map
*R*[*F*^2^ > 2σ(*F*^2^)] = 0.026	Hydrogen site location: inferred from neighbouring sites
*wR*(*F*^2^) = 0.086	H atoms treated by a mixture of independent and constrained refinement
*S* = 1.10	*w* = 1/[σ^2^(*F*_o_^2^) + (0.0381*P*)^2^ + 3.2233*P*] where *P* = (*F*_o_^2^ + 2*F*_c_^2^)/3
2617 reflections	(Δ/σ)_max_ = 0.001
223 parameters	Δρ_max_ = 0.82 e Å^−^^3^
2 restraints	Δρ_min_ = −0.74 e Å^−^^3^

### Special details

**Table:**

Geometry. All e.s.d.'s (except the e.s.d. in the dihedral angle between two l.s. planes) are estimated using the full covariance matrix. The cell e.s.d.'s are taken into account individually in the estimation of e.s.d.'s in distances, angles and torsion angles; correlations between e.s.d.'s in cell parameters are only used when they are defined by crystal symmetry. An approximate (isotropic) treatment of cell e.s.d.'s is used for estimating e.s.d.'s involving l.s. planes.
Refinement. Refinement of *F*^2^ against ALL reflections. The weighted *R*-factor *wR* and goodness of fit *S* are based on *F*^2^, conventional *R*-factors *R* are based on *F*, with *F* set to zero for negative *F*^2^. The threshold expression of *F*^2^ > σ(*F*^2^) is used only for calculating *R*-factors(gt) *etc*. and is not relevant to the choice of reflections for refinement. *R*-factors based on *F*^2^ are statistically about twice as large as those based on *F*, and *R*- factors based on ALL data will be even larger.

### Fractional atomic coordinates and isotropic or equivalent isotropic displacement parameters (Å^2^)

**Table:**

	*x*	*y*	*z*	*U*_iso_*/*U*_eq_	
Cd1	0.04544 (4)	0.84510 (4)	0.07477 (3)	0.01071 (12)	
N2	0.2174 (5)	1.0166 (4)	0.2915 (4)	0.0132 (8)	
N1	−0.0501 (5)	0.7203 (4)	0.2200 (4)	0.0142 (8)	
C5	−0.1372 (7)	0.5878 (6)	0.4074 (5)	0.0177 (10)	
H5	−0.1662	0.5412	0.4702	0.021*	
C14	0.3403 (6)	1.1629 (5)	0.3271 (4)	0.0153 (9)	
H14	0.3565	1.2070	0.2604	0.018*	
C3	−0.1898 (7)	0.5807 (5)	0.1841 (5)	0.0174 (10)	
H3	−0.2601	0.5262	0.0933	0.021*	
C13	0.4480 (7)	1.2563 (5)	0.4588 (5)	0.0178 (10)	
H13	0.5356	1.3604	0.4803	0.021*	
C6	0.0091 (7)	0.7368 (6)	0.4475 (4)	0.0155 (9)	
C7	0.0472 (6)	0.7983 (5)	0.3497 (4)	0.0123 (9)	
C11	0.1893 (6)	0.9555 (5)	0.3877 (4)	0.0131 (9)	
C8	0.1133 (7)	0.8288 (6)	0.5828 (4)	0.0180 (10)	
H8	0.0885	0.7863	0.6487	0.022*	
C9	0.2449 (7)	0.9734 (6)	0.6180 (5)	0.0200 (10)	
H9	0.3107	1.0318	0.7084	0.024*	
C10	0.2890 (6)	1.0423 (5)	0.5221 (4)	0.0141 (9)	
C12	0.4230 (7)	1.1927 (5)	0.5559 (5)	0.0187 (10)	
H12	0.4973	1.2515	0.6454	0.022*	
C4	−0.2371 (7)	0.5112 (6)	0.2757 (5)	0.0197 (10)	
H4	−0.3380	0.4114	0.2467	0.024*	
O1W	0.2864 (5)	0.7375 (4)	0.1138 (3)	0.0147 (7)	
O4	0.0112 (4)	0.7484 (4)	−0.1567 (3)	0.0166 (7)	
O3	−0.1556 (5)	0.5788 (4)	−0.0899 (3)	0.0193 (7)	
N3	−0.1132 (5)	0.6148 (4)	−0.1844 (4)	0.0129 (8)	
O5	−0.1930 (5)	0.5236 (4)	−0.2976 (3)	0.0226 (8)	
O1	−0.1906 (4)	0.9255 (4)	−0.0170 (3)	0.0141 (6)	
O2	−0.3962 (5)	0.7674 (4)	0.0468 (3)	0.0204 (7)	
C1	−0.3553 (6)	0.8680 (5)	−0.0059 (4)	0.0103 (8)	
C2	−0.5043 (6)	0.9368 (5)	−0.0573 (4)	0.0118 (9)	
H2A	−0.6357	0.8539	−0.0978	0.014*	
H2B	−0.4758	0.9823	−0.1247	0.014*	
H1B	0.238 (7)	0.6402 (19)	0.106 (5)	0.018*	
H1A	0.373 (5)	0.736 (6)	0.080 (5)	0.018*	

### Atomic displacement parameters (Å^2^)

**Table:**

	*U*^11^	*U*^22^	*U*^33^	*U*^12^	*U*^13^	*U*^23^
Cd1	0.01089 (18)	0.01093 (18)	0.00981 (18)	0.00348 (12)	0.00347 (12)	0.00352 (12)
N2	0.0124 (18)	0.016 (2)	0.0127 (18)	0.0055 (16)	0.0053 (15)	0.0070 (15)
N1	0.0144 (19)	0.0113 (19)	0.0161 (19)	0.0050 (15)	0.0048 (15)	0.0032 (15)
C5	0.023 (3)	0.019 (2)	0.020 (2)	0.010 (2)	0.014 (2)	0.012 (2)
C14	0.014 (2)	0.020 (2)	0.015 (2)	0.0075 (19)	0.0068 (18)	0.0064 (18)
C3	0.019 (2)	0.012 (2)	0.019 (2)	0.0028 (19)	0.0079 (19)	0.0040 (18)
C13	0.015 (2)	0.007 (2)	0.026 (3)	−0.0007 (18)	0.008 (2)	0.0010 (19)
C6	0.020 (2)	0.021 (2)	0.016 (2)	0.013 (2)	0.0106 (19)	0.0131 (19)
C7	0.011 (2)	0.015 (2)	0.014 (2)	0.0058 (18)	0.0053 (17)	0.0076 (18)
C11	0.015 (2)	0.020 (2)	0.011 (2)	0.0110 (19)	0.0081 (17)	0.0057 (18)
C8	0.023 (2)	0.027 (3)	0.013 (2)	0.014 (2)	0.0093 (19)	0.012 (2)
C9	0.020 (2)	0.027 (3)	0.014 (2)	0.011 (2)	0.0046 (19)	0.006 (2)
C10	0.015 (2)	0.014 (2)	0.014 (2)	0.0082 (18)	0.0056 (18)	0.0013 (18)
C12	0.016 (2)	0.015 (2)	0.018 (2)	0.0037 (19)	0.0029 (19)	−0.0013 (19)
C4	0.019 (2)	0.017 (2)	0.023 (3)	0.006 (2)	0.011 (2)	0.003 (2)
O1W	0.0139 (16)	0.0115 (16)	0.0203 (17)	0.0042 (13)	0.0083 (13)	0.0062 (13)
O4	0.0154 (16)	0.0109 (16)	0.0195 (17)	−0.0002 (13)	0.0063 (13)	0.0044 (13)
O3	0.0243 (18)	0.0210 (18)	0.0138 (16)	0.0057 (15)	0.0113 (14)	0.0062 (14)
N3	0.0138 (19)	0.0103 (19)	0.0136 (19)	0.0047 (15)	0.0048 (15)	0.0019 (15)
O5	0.0228 (18)	0.0244 (19)	0.0096 (16)	0.0025 (15)	0.0019 (14)	−0.0015 (14)
O1	0.0081 (15)	0.0195 (17)	0.0137 (15)	0.0036 (13)	0.0037 (12)	0.0057 (13)
O2	0.0181 (17)	0.0206 (18)	0.036 (2)	0.0104 (14)	0.0169 (15)	0.0219 (16)
C1	0.015 (2)	0.006 (2)	0.011 (2)	0.0038 (17)	0.0065 (17)	0.0012 (16)
C2	0.011 (2)	0.011 (2)	0.015 (2)	0.0026 (17)	0.0047 (17)	0.0082 (18)

### Geometric parameters (Å, º)

**Table:**

Cd1—O1	2.253 (3)	C7—C11	1.453 (6)
Cd1—O1W	2.355 (3)	C11—C10	1.414 (6)
Cd1—N1	2.358 (4)	C8—C9	1.340 (7)
Cd1—N2	2.360 (4)	C8—H8	0.9500
Cd1—O1^i^	2.439 (3)	C9—C10	1.437 (7)
Cd1—O4	2.470 (3)	C9—H9	0.9500
Cd1—O3	2.511 (3)	C10—C12	1.389 (7)
N2—C14	1.327 (6)	C12—H12	0.9500
N2—C11	1.365 (6)	C4—H4	0.9500
N1—C3	1.330 (6)	O1W—H1B	0.859 (10)
N1—C7	1.355 (6)	O1W—H1A	0.859 (10)
C5—C4	1.374 (7)	O4—N3	1.254 (5)
C5—C6	1.407 (7)	O3—N3	1.275 (5)
C5—H5	0.9500	N3—O5	1.234 (5)
C14—C13	1.410 (6)	O1—C1	1.282 (5)
C14—H14	0.9500	O1—Cd1^i^	2.439 (3)
C3—C4	1.405 (7)	O2—C1	1.232 (5)
C3—H3	0.9500	C1—C2	1.521 (6)
C13—C12	1.381 (7)	C2—C2^ii^	1.528 (9)
C13—H13	0.9500	C2—H2A	0.9900
C6—C7	1.406 (6)	C2—H2B	0.9900
C6—C8	1.435 (7)		
			
O1—Cd1—O1W	165.04 (11)	N1—C7—C6	122.8 (4)
O1—Cd1—N1	107.49 (12)	N1—C7—C11	117.9 (4)
O1W—Cd1—N1	83.07 (12)	C6—C7—C11	119.3 (4)
O1—Cd1—N2	107.06 (12)	N2—C11—C10	122.0 (4)
O1W—Cd1—N2	86.20 (12)	N2—C11—C7	118.5 (4)
N1—Cd1—N2	70.97 (13)	C10—C11—C7	119.4 (4)
O1—Cd1—O1^i^	71.54 (12)	C9—C8—C6	121.2 (4)
O1W—Cd1—O1^i^	103.94 (10)	C9—C8—H8	119.4
N1—Cd1—O1^i^	153.21 (12)	C6—C8—H8	119.4
N2—Cd1—O1^i^	83.58 (11)	C8—C9—C10	121.5 (4)
O1—Cd1—O4	81.10 (11)	C8—C9—H9	119.2
O1W—Cd1—O4	83.94 (11)	C10—C9—H9	119.2
N1—Cd1—O4	133.14 (12)	C12—C10—C11	118.3 (4)
N2—Cd1—O4	152.10 (12)	C12—C10—C9	122.7 (4)
O1^i^—Cd1—O4	73.64 (10)	C11—C10—C9	119.0 (4)
O1—Cd1—O3	87.96 (11)	C13—C12—C10	119.8 (4)
O1W—Cd1—O3	82.85 (11)	C13—C12—H12	120.1
N1—Cd1—O3	82.58 (12)	C10—C12—H12	120.1
N2—Cd1—O3	152.40 (12)	C5—C4—C3	119.9 (4)
O1^i^—Cd1—O3	123.65 (10)	C5—C4—H4	120.1
O4—Cd1—O3	51.17 (10)	C3—C4—H4	120.1
C14—N2—C11	118.1 (4)	Cd1—O1W—H1B	112 (4)
C14—N2—Cd1	126.2 (3)	Cd1—O1W—H1A	132 (4)
C11—N2—Cd1	115.7 (3)	H1B—O1W—H1A	99 (5)
C3—N1—C7	118.5 (4)	N3—O4—Cd1	97.2 (2)
C3—N1—Cd1	125.0 (3)	N3—O3—Cd1	94.7 (2)
C7—N1—Cd1	116.5 (3)	O5—N3—O4	121.9 (4)
C4—C5—C6	118.8 (4)	O5—N3—O3	121.5 (4)
C4—C5—H5	120.6	O4—N3—O3	116.6 (3)
C6—C5—H5	120.6	C1—O1—Cd1	117.6 (3)
N2—C14—C13	123.4 (4)	C1—O1—Cd1^i^	132.2 (3)
N2—C14—H14	118.3	Cd1—O1—Cd1^i^	108.46 (12)
C13—C14—H14	118.3	O2—C1—O1	124.8 (4)
N1—C3—C4	122.3 (4)	O2—C1—C2	119.1 (4)
N1—C3—H3	118.9	O1—C1—C2	116.0 (4)
C4—C3—H3	118.9	C1—C2—C2^ii^	108.6 (4)
C12—C13—C14	118.3 (4)	C1—C2—H2A	110.0
C12—C13—H13	120.8	C2^ii^—C2—H2A	110.0
C14—C13—H13	120.8	C1—C2—H2B	110.0
C7—C6—C5	117.9 (4)	C2^ii^—C2—H2B	110.0
C7—C6—C8	119.6 (4)	H2A—C2—H2B	108.4
C5—C6—C8	122.5 (4)		
			
O1—Cd1—N2—C14	−73.4 (4)	C5—C6—C8—C9	−176.6 (5)
O1W—Cd1—N2—C14	99.5 (4)	C6—C8—C9—C10	−0.6 (7)
N1—Cd1—N2—C14	−176.5 (4)	N2—C11—C10—C12	−1.8 (6)
O1^i^—Cd1—N2—C14	−5.0 (3)	C7—C11—C10—C12	−179.5 (4)
O4—Cd1—N2—C14	30.1 (5)	N2—C11—C10—C9	176.9 (4)
O3—Cd1—N2—C14	166.2 (3)	C7—C11—C10—C9	−0.9 (6)
O1—Cd1—N2—C11	108.4 (3)	C8—C9—C10—C12	179.2 (4)
O1W—Cd1—N2—C11	−78.7 (3)	C8—C9—C10—C11	0.6 (7)
N1—Cd1—N2—C11	5.3 (3)	C14—C13—C12—C10	−2.4 (7)
O1^i^—Cd1—N2—C11	176.8 (3)	C11—C10—C12—C13	3.5 (7)
O4—Cd1—N2—C11	−148.1 (3)	C9—C10—C12—C13	−175.1 (4)
O3—Cd1—N2—C11	−12.0 (5)	C6—C5—C4—C3	1.2 (7)
O1—Cd1—N1—C3	72.5 (4)	N1—C3—C4—C5	−0.1 (7)
O1W—Cd1—N1—C3	−96.7 (4)	O1—Cd1—O4—N3	−91.1 (2)
N2—Cd1—N1—C3	175.0 (4)	O1W—Cd1—O4—N3	89.2 (2)
O1^i^—Cd1—N1—C3	156.0 (3)	N1—Cd1—O4—N3	14.6 (3)
O4—Cd1—N1—C3	−21.7 (4)	N2—Cd1—O4—N3	159.1 (3)
O3—Cd1—N1—C3	−13.0 (4)	O1^i^—Cd1—O4—N3	−164.3 (3)
O1—Cd1—N1—C7	−107.8 (3)	O3—Cd1—O4—N3	3.5 (2)
O1W—Cd1—N1—C7	83.1 (3)	O1—Cd1—O3—N3	76.8 (2)
N2—Cd1—N1—C7	−5.3 (3)	O1W—Cd1—O3—N3	−91.4 (2)
O1^i^—Cd1—N1—C7	−24.3 (5)	N1—Cd1—O3—N3	−175.3 (3)
O4—Cd1—N1—C7	158.0 (3)	N2—Cd1—O3—N3	−158.8 (3)
O3—Cd1—N1—C7	166.7 (3)	O1^i^—Cd1—O3—N3	10.6 (3)
C11—N2—C14—C13	2.3 (6)	O4—Cd1—O3—N3	−3.4 (2)
Cd1—N2—C14—C13	−175.8 (3)	Cd1—O4—N3—O5	172.9 (3)
C7—N1—C3—C4	−1.1 (7)	Cd1—O4—N3—O3	−6.0 (4)
Cd1—N1—C3—C4	178.6 (3)	Cd1—O3—N3—O5	−173.1 (3)
N2—C14—C13—C12	−0.6 (7)	Cd1—O3—N3—O4	5.9 (4)
C4—C5—C6—C7	−1.0 (7)	O1W—Cd1—O1—C1	118.5 (4)
C4—C5—C6—C8	176.5 (4)	N1—Cd1—O1—C1	−15.1 (3)
C3—N1—C7—C6	1.3 (6)	N2—Cd1—O1—C1	−90.0 (3)
Cd1—N1—C7—C6	−178.5 (3)	O1^i^—Cd1—O1—C1	−166.9 (4)
C3—N1—C7—C11	−175.4 (4)	O4—Cd1—O1—C1	117.4 (3)
Cd1—N1—C7—C11	4.8 (5)	O3—Cd1—O1—C1	66.4 (3)
C5—C6—C7—N1	−0.2 (7)	O1W—Cd1—O1—Cd1^i^	−74.6 (4)
C8—C6—C7—N1	−177.8 (4)	N1—Cd1—O1—Cd1^i^	151.83 (13)
C5—C6—C7—C11	176.4 (4)	N2—Cd1—O1—Cd1^i^	76.95 (14)
C8—C6—C7—C11	−1.1 (6)	O1^i^—Cd1—O1—Cd1^i^	0.0
C14—N2—C11—C10	−1.1 (6)	O4—Cd1—O1—Cd1^i^	−75.62 (12)
Cd1—N2—C11—C10	177.3 (3)	O3—Cd1—O1—Cd1^i^	−126.61 (12)
C14—N2—C11—C7	176.7 (4)	Cd1—O1—C1—O2	−2.9 (6)
Cd1—N2—C11—C7	−5.0 (5)	Cd1^i^—O1—C1—O2	−166.1 (3)
N1—C7—C11—N2	0.2 (6)	Cd1—O1—C1—C2	174.2 (3)
C6—C7—C11—N2	−176.7 (4)	Cd1^i^—O1—C1—C2	11.0 (5)
N1—C7—C11—C10	178.0 (4)	O2—C1—C2—C2^ii^	81.3 (6)
C6—C7—C11—C10	1.1 (6)	O1—C1—C2—C2^ii^	−96.0 (5)
C7—C6—C8—C9	0.8 (7)		

Symmetry codes: (i) −*x*, −*y*+2, −*z*; (ii) −*x*−1, −*y*+2, −*z*.

### Hydrogen-bond geometry (Å, º)

**Table:**

*D*—H···*A*	*D*—H	H···*A*	*D*···*A*	*D*—H···*A*
O1*W*—H1*B*···O3^iii^	0.86 (1)	1.93 (1)	2.786 (5)	174 (5)
O1*W*—H1*A*···O2^iv^	0.86 (1)	1.88 (2)	2.716 (4)	166 (5)

Symmetry codes: (iii) −*x*, −*y*+1, −*z*; (iv) *x*+1, *y*, *z*.
